# Monoamine oxidase A is down-regulated in EBV-associated nasopharyngeal carcinoma

**DOI:** 10.1038/s41598-020-63150-0

**Published:** 2020-04-09

**Authors:** Hui Min Lee, Alice Pei Eal Sia, Lili Li, Hans Prakash Sathasivam, Melissa Sue Ann Chan, Pathmanathan Rajadurai, Chi Man Tsang, Sai Wah Tsao, Paul G. Murray, Qian Tao, Ian C. Paterson, Lee Fah Yap

**Affiliations:** 1 Department of Oral and Craniofacial Sciences, Faculty of Dentistry, University of Malaya, Kuala Lumpur, Malaysia; 2 Cancer Epigenetics Laboratory, Department of Clinical Oncology, State Key Laboratory of Translational Oncology, Sir YK Pao Center for Cancer and Li Ka Shing Institute of Health Sciences, The Chinese University of Hong Kong, Shatin, Hong Kong; 3 Cancer Research Centre, Institute for Medical Research, Shah Alam, Malaysia; 4 Sime Darby Medical Centre Subang Jaya, Subang Jaya, Malaysia; 5 School of Biomedical Sciences and Center for Cancer Research, Li Ka Shing Faculty of Medicine, The University of Hong Kong, Shatin, Hong Kong; 6 Department of Anatomical and Cellular Pathology and State Key Laboratory of Translational Oncology, The Chinese University of Hong Kong, Pokfulam, Hong Kong; 7 Health Research Institute, University of Limerick, Limerick, Ireland; 8 Institute of Immunology and Immunotherapy, University of Birmingham, Birmingham, United Kingdom; 9 Oral Cancer Research and Coordinating Centre, Faculty of Dentistry, University of Malaya, Kuala Lumpur, Malaysia

**Keywords:** Head and neck cancer, Tumour-suppressor proteins, Tumour virus infections, Medical research

## Abstract

Nasopharyngeal carcinoma (NPC) is a highly metastatic cancer that is consistently associated with Epstein-Barr virus (EBV) infection. In this study, we identify for the first time a role for monoamine oxidase A (MAOA) in NPC. MAOA is a mitochondrial enzyme that catalyzes oxidative deamination of neurotransmitters and dietary amines. Depending on the cancer type, MAOA can either have a tumour-promoting or tumour-suppressive role. We show that MAOA is down-regulated in primary NPC tissues and its down-regulation enhances the migration of NPC cells. In addition, we found that EBV infection can down-regulate MAOA expression in both pre-malignant and malignant nasopharyngeal epithelial (NPE) cells. We further demonstrate that MAOA is down-regulated as a result of IL-6/IL-6R/STAT3 signalling and epigenetic mechanisms, effects that might be attributed to EBV infection in NPE cells. Taken together, our data point to a central role for EBV in mediating the tumour suppressive effects of MAOA and that loss of MAOA could be an important step in the pathogenesis of NPC.

## Introduction

Nasopharyngeal carcinoma (NPC) is a cancer with high invasive potential which is exceptionally common in Southern China and Southeast Asia^1,2^. Delayed presentation is a major challenge in NPC management with>75% of patients present with late stage disease^1^. A significant proportion of these patients (approximately 30%) develop distant metastases post therapy, which represents the major cause of death for NPC patients^1,3^. The mainstay of current treatment for advanced disease is restricted to concurrent chemoradiotherapy and regrettably, many NPC survivors suffer from an impaired health-related quality of life because the tumours are located in close proximity to many vital organs in the head and neck region^4,5^. Therefore, a better understanding of the molecular pathogenesis of NPC is essential to inform therapeutic interventions.

Undifferentiated type of NPC is invariably associated with Epstein-Barr virus (EBV) infection^6^. The impact of EBV infection on the development of NPC is thought to be a consequence of the aberrant establishment of virus latency in nasopharyngeal epithelial (NPE) cells displaying pre-malignant genetic changes (possibly as a result of exposure to environmental insults). Following infection, EBV induces additional changes resulting in the development of NPC. EBV latent gene expression in NPC is commonly restricted to Epstein-Barr nuclear antigen 1 (EBNA1), latent membrane proteins (LMP 1 and LMP2), EBV-encoded RNAs (EBERs) and *Bam*HI-A transcripts. It is well established that many of the oncogenic effects of EBV in NPE cells can be attributed to its latent genes/proteins^6^. Although the association between EBV and NPC has been well-established, the exact contribution of EBV to the cellular gene expression programme of NPC remains to be fully elucidated.

Monoamine oxidase A (MAOA) is a mitochondrial enzyme that catalyzes the oxidative deamination of monoamine neurotransmitters (serotonin, norepinephrine and dopamine) and dietary amines (tyramine)^7,8^. The function of MAOA is well-defined in neurological disorders but its role in carcinogenesis appears to be more diverse^9^. An early study described the down-regulation of MAOA in multiple human cancer types^10^ and subsequently, a tumour-suppressive role of MAOA was reported in esophageal cancer^11^, cholangiocarcinoma^12^, hepatocellular carcinoma^13^ and breast cancer^14^. Conversely, tumour-promoting effects of MAOA have been described in prostate cancer^15–18^, and its overexpression was also reported in renal cell carcinoma^19^, glioma^20^ and non-small cell lung cancer^21^. Notably, MAOA was shown to be highly expressed in classical Hodgkin lymphoma (cHL), with expression more commonly seen in EBV-negative compared with EBV-positive cHL, especially in the EBV-negative nodular sclerosis subtype^22^. Here, we show for the first time that EBV infection down-regulates the expression of MAOA in pre-malignant and malignant NPE cells, possibly in part through IL-6/IL-6R signalling as well as epigenetic mechanisms. Importantly, we show that the down-regulation of MAOA enhances NPC cell migration. Taken together, our data provide evidence of a tumour-suppressive function for MAOA in EBV-associated NPC.

## Results

### Down-regulation of MAOA expression in NPC

**Figure 1:**
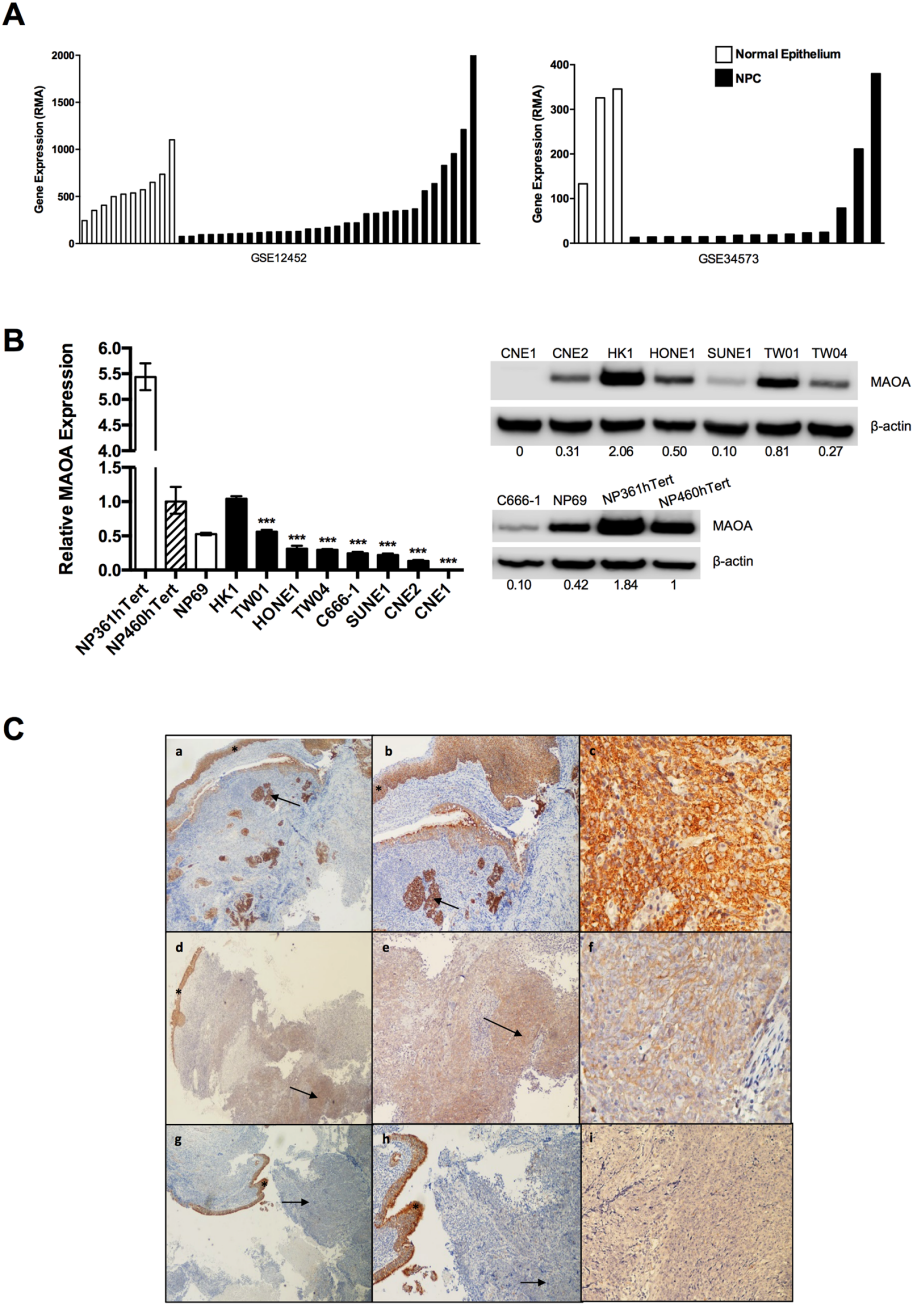
MAOA is down-regulated in NPC. (**A**) Two microarray datasets (GSE12452 and GSE34573) showed that MAOA mRNA was significantly down-regulated in micro-dissected NPC cells compared with normal epithelium (p < 0.01). (**B**) Compared to NP460hTert, MAOA mRNA and protein levels were decreased in seven (C666-1, CNE1, CNE2, HONE1, SUNE1, TW01 and TW04) out of eight NPC cell lines examined. Densitometric data are expressed as the relative density (normalized to β-actin). Full-length blots are presented in Supplementary Fig. S1. ***p < 0.001. (**C**) Immunohistochemical analysis of MAOA expression in primary NPC. (a,b): A case demonstrating positive MAOA staining. The adjacent normal epithelium (asterisk) and underlying islands of carcinoma (arrow) are both strongly positive; (c) High-power view of area highlighted by arrow in b. (d,e): A case demonstrating moderately positive MAOA staining of carcinoma (arrow) beneath the overlying epithelium (asterisk) that is strongly positive; (f) High-power view of area highlighted by arrow in e. (g,h): A case demonstrating negative MAOA staining. The adjacent normal epithelium (asterisk) is strongly positive whilst there is no staining in the carcinoma (arrow); (i) High-power view of area highlighted by arrow in h. Original magnification (a,d,g) ×40; (b,e,h) ×100; (c,f,i) ×400.

Re-analysis of two published microarray datasets, GSE12452^23^ and GSE34573^24^, revealed significant down-regulation of MAOA mRNA expression in the majority of micro-dissected NPC cells compared with normal epithelium (p < 0.01; Fig. 1A). MAOA mRNA expression in eight NPC cell lines (CNE1, CNE2, C666-1, HK1, HONE1, SUNE1, TW01 and TW04) was compared with that in three immortalised NPE cell lines, NP69, NP361hTert and NP460hTert. Compared to NP460hTert which is commonly used as a representative pre-malignant NPE cell line, seven NPC cell lines (CNE1, CNE2, C666-1, HONE1, SUNE1, TW01 and TW04) showed reduced levels of MAOA at both mRNA and protein levels (Fig. 1B). The expression and cellular localization of MAOA protein were then examined in archival formalin-fixed paraffin-embedded (FFPE) NPC (n = 17) and non-cancerous nasopharyngeal tissues (n = 3) using immunohistochemistry. As shown in Fig. 1C, MAOA expression was significantly reduced in NPC (mean H-score of 91, range 0–290, mode 0, p < 0.0001) compared to normal and non-dysplastic surface epithelia (mean H-score of 278, range 190–300, mode 300). In 8 of 17 (47%) tumours, the H-score was 0. We conclude that MAOA is down-regulated in a subset of NPC.

### MAOA inhibits NPC cell migration

**Figure 2:**
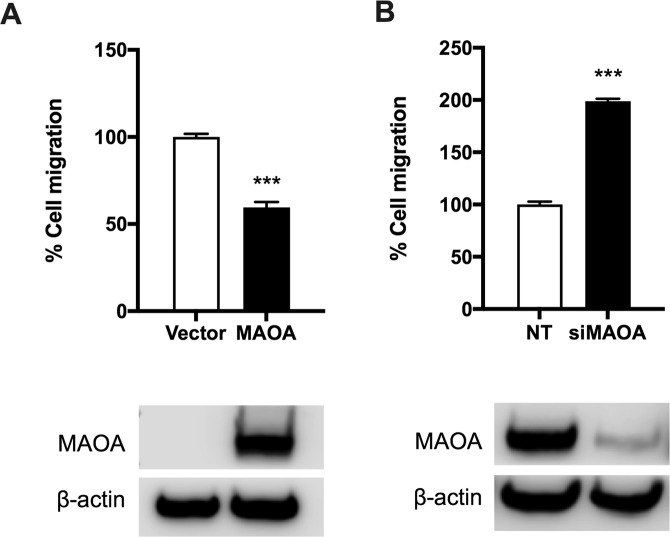
MAOA inhibits NPC cell migration. (**A**) Ectopic expression of MAOA significantly suppressed the migration of SUNE1 cells. Increased expression of MAOA was confirmed by Western blotting. (**B**) Inhibition of MAOA significantly enhanced the migration of HONE1 cells. Reduced levels of MAOA following siRNA treatment was confirmed by Western blotting. Full-length blots are presented in Supplementary Fig. S1. ***p < 0.001.

We next investigated if MAOA influences NPC cell behaviour *in vitro*, focussing on the migratory phenotype because distant metastasis remains a major cause of death for NPC patients. We used Transwell assays to examine the function of MAOA on the migration of NPC cells using two representative NPC cell lines (SUNE1 and HONE1). These cell lines were chosen based on the low endogenous levels of MAOA in SUNE1 and detectable levels of MAOA in HONE1. SUNE1 cells were transduced with pEZ-lv105/MAOA or with the empty vector, and the increased expression of MAOA protein was confirmed (Fig. 2A). The results showed that ectopic expression of MAOA significantly suppressed the migration of SUNE1 cells (p < 0.001; Fig. 2A). A complementary approach was performed in which MAOA was knocked down using siRNA in HONE1 cells and the reduced expression of MAOA protein was confirmed (Fig. 2B). In line with the data obtained from the ectopic expression experiments in SUNE1, knockdown of MAOA significantly increased the migration of HONE1 cells (p < 0.001; Fig. 2B).

### EBV infection of NPE cells decreases the expression of MAOA

**Figure 3:**
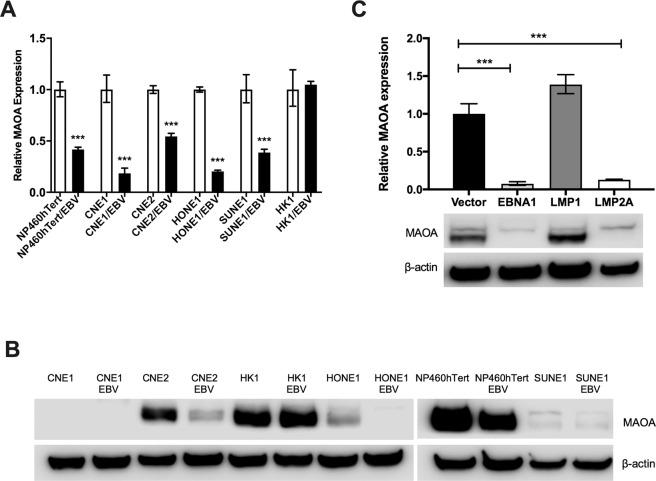
EBV infection down-regulates the expression of MAOA. (**A**) RT-qPCR analysis showed that the expression of MAOA was significantly reduced in an immortalised NPE cell line (NP460hTert) and four (CNE1, CNE2, HONE1, SUNE1) NPC cell lines stably infected with an Akata-derived recombinant EBV. Data are expressed as the relative expression between the cells infected with EBV and their respective controls (normalised to 1). (**B**) Western blot analysis showed that the protein levels of MAOA were reduced in NP460hTert and two NPC cell lines (CNE2 and HONE1) following EBV infection. (**C**) RT-qPCR and Western blotting analyses showed that both the mRNA and protein levels of MAOA were lower in HONE1 cells expressing specific EBV latent genes (EBNA1 or LMP2A) compared to the vector control cells. Full-length blots are presented in Supplementary Fig. S1. ***p < 0.001.

Having shown that MAOA is down-regulated in NPC, we opted to investigate whether EBV infection could modulate MAOA levels in NPC cells. We first examined MAOA mRNA expression in six cell lines (NP460hTert, CNE1, CNE2, HK1, HONE1 and SUNE1) stably infected with a recombinant EBV (Akata strain). We found that the levels of MAOA mRNA were significantly decreased in five cell lines (NP460hTert, CNE1, CNE2, HONE1 and SUNE1) infected with EBV with the exception of HK1 which was derived from a well-differentiated NPC tumour (p < 0.001; Fig. 3A). Among these cell lines, the reduction of MAOA protein expression was also evident in three EBV-infected cell lines (CNE2, HONE1 and NP460hTert) at the protein level (Fig. 3B). Despite being expressed at the mRNA level, MAOA protein was not detected in CNE1 and SUNE1 (Fig. 3B). Next, we investigated which EBV gene(s) were responsible for this effect. To do this, we examined MAOA expression in HONE1 cells individually transfected with the EBV genes, EBNA1, LMP1 or LMP2A, all of which are expressed in NPC *in vivo*. The expression of these genes in HONE1 cells was confirmed by RT-qPCR (Supplementary Fig. 2). We found that both the mRNA and protein levels of MAOA were down-regulated by EBNA1 and LMP2A, but not by LMP1, in HONE1 cells (Fig. 3C). These data indicate that EBV infection can reduce MAOA levels in both pre-malignant and malignant NPE cells *in vitro*.

### IL-6/IL-6R/STAT3 inhibits MAOA expression

**Figure 4:**
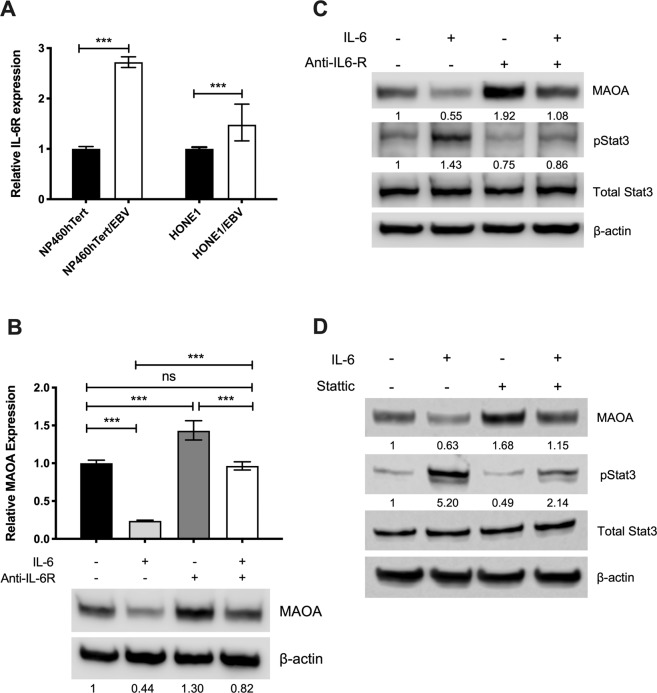
IL-6/IL-6R suppresses MAOA expression through STAT3. (**A**) Increased expression of IL-6R in NP460hTert and HONE1 cells following EBV infection. (**B**) Both the mRNA and protein levels of MAOA were decreased in NP460hTert/EBV following addition of IL-6 (50 ng/ml) and these effects were rescued by blocking IL-6R using an anti-IL-6R neutralizing antibody (1 µg/ml). (**C**) Following the addition of IL-6 in NP460hTert/EBV, the decrease in MAOA levels was accompanied by the activation of STAT3 and the expression of MAOA was restored with concomitant inhibition of phosphorylated STAT3 following the treatment with an IL-6R neutralising antibody. (**D**) Treatment of NP460hTert with a STAT3 inhibitor, Stattic, restored the expression of MAOA. Densitometric data are expressed as the relative density (MAOA: normalized to β-actin; pSTAT3: normalized to total STAT3). Full-length blots are presented in Supplementary Fig. S1. ***p < 0.001.

Having shown that EBV infection can reduce MAOA levels in NPC cells, we then investigated the mechanism of its down-regulation if this might be attributed to EBV infection. We focussed on the IL-6/IL-6R signalling because this pathway has been shown to inhibit MAOA expression in other cancer types^12,14^ and secondly, activation of this pathway has been reported in EBV-infected NPE cells and has been shown to promote their malignant properties, including migration^25^. In agreement with a previous report^25^, we showed that IL-6R mRNA expression was significantly increased in NP460hTert and HONE1 cells following EBV infection (p < 0.001; Fig. 4A). We investigated whether IL-6/IL-6R signalling can modulate MAOA expression and we found that treatment of NP460hTert/EBV cells with IL-6 significantly decreased the expression of MAOA (p < 0.001; Fig. 4B), an effect that was rescued by blocking the IL-6R (Fig. 4B). We next determined if the down-regulation of MAOA induced by IL-6/IL-6R signalling was mediated through STAT3. Activation of STAT3 was accompanied by the decreased MAOA expression in NP460hTert/EBV cells treated with IL-6 and the expression of MAOA was restored with concomitant inhibition of STAT3 phosphorylation following treatment with an IL-6R neutralising antibody (Fig. 4C). To further confirm the specificity of these effects, the cells were treated with a STAT3 inhibitor, Stattic, which also restored the expression of MAOA in NP460hTert/EBV cells (Fig. 4D). We conclude that IL-6/IL-6R/STAT3 can down-regulate MAOA expression and these effects could be attributed to EBV infection in NPE cells.

### MAOA is down-regulated in NPC partly through epigenetic silencing

**Figure 5:**
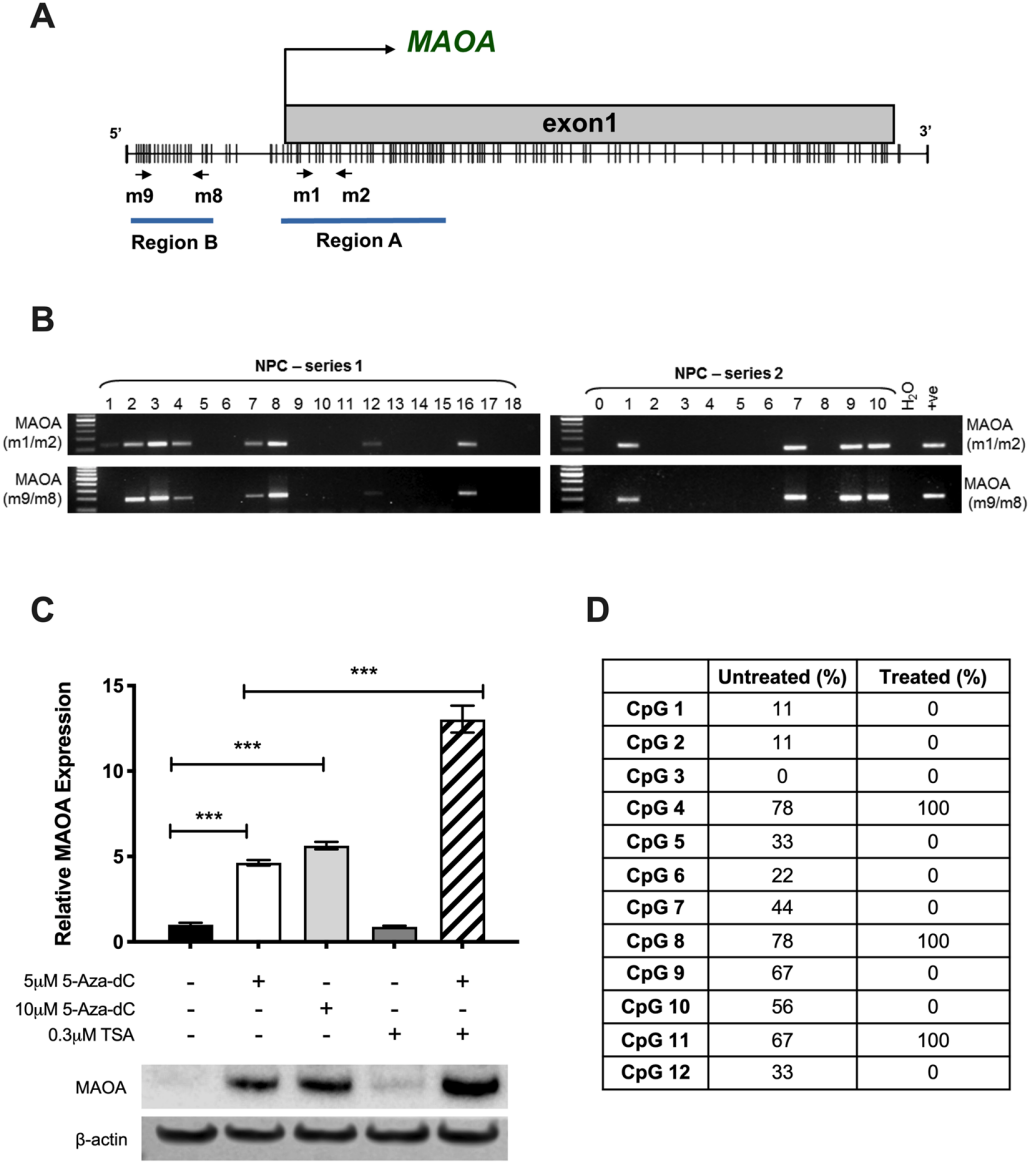
MAOA is down-regulated partly by epigenetic silencing. (**A**) A schematic diagram showing MAOA promoter region. Two pairs of MSP primers (m1/m2 and m9/m8) targeting different regions were designed. (**B**) MSP analyses using two different pairs of primers yielded similar results and methylation of MAOA promoter was detected in primary NPC tissues examined. Representative results are presented. Full-length gels are presented in Supplementary Fig. S1. (**C**) RT-qPCR and Western blotting analyses showed that treatment of SUNE1 cells with 5-Aza-dC restored the expression of MAOA and the levels were further increased in the presence of TSA. ***p < 0.001. Full-length blots are presented in Supplementary Fig. S1. (**D**) Bisulfite sequencing analysis showed that the degree of methylation in the promoter of 5-Aza-dC/TSA-treated SUNE1 cells was generally lower than that in the untreated control.

Given that EBV infection induces DNA methylation in malignant epithelial cells^26^ and there is evidence showing that MAOA expression can be epigenetically regulated^12^, we next examined the methylation status of the MAOA promoter in NPC. The MAOA promoter region is a typical CpG island, and two pairs of MSP primers (m1/m2 and m9/m8) targeting different regions were designed (Fig. 5A). We used DNAs from a series of 54 primary NPC tissues which we have shown previously to be of good quality and suitable for MSP analyses^27–30^. MSP analyses using two different pairs of primers yielded similar results and methylation of the MAOA promoter was detected in 22 of 54 (40.7%) primary NPC tissues examined (Fig. 5B). In support of these data, the expression of MAOA was restored in SUNE1 cells following the treatment with 5-aza-dC in the presence or absence of TSA (Fig. 5C). BSP analysis demonstrated that the degree of methylation in the promoter of treated SUNE1 cells was generally lower than that in the untreated control (Fig. 5D). These findings indicate that epigenetic silencing is one of the mechanisms responsible for the low levels of MAOA expression in NPC.

## Discussion

There is evidence to suggest that neurotransmitters are key players in the regulation of tumour cell migration, similar to the well-established effect of chemokines^31^. For example, it has been shown that norepinephrine and dopamine (substrates of MAOA) induced chemotaxis in breast cancer cells^32^. Although the role of such neurotransmitters in NPC is currently unknown, our results that show MAOA down-regulation promoting NPC cell migration supports these observations. Similarly, the reduced expression of MAOA has been shown to promote an invasive phenotype in cholangiocarcinoma, hepatocellular carcinoma and breast cancer^12–14^. Conversely, overexpression of MAOA was reported in other cancer types, such as renal cell carcinoma, glioma, cHL, non-small cell lung cancer, and its oncogenic role is particularly well-described in prostate cancer, involving a ROS-activated AKT/FOXO/TWIST1 signalling pathway and maintenance of cancer stem cells^9,19–22^. Interestingly, a link between MAOA expression and EBV infection was implied by data showing that its expression was more commonly seen in EBV-negative than EBV-positive cHL and was particularly prevalent in the EBV-negative nodular sclerosis subtype^22^. In support of these observations, here we provide the first evidence to show that EBV infection can down-regulate MAOA in pre-malignant, as well as malignant NPE cells. We also noted that the expression of MAOA in HK1 cells (derived from a well-differentiated EBV-negative NPC tumour) was comparable to that of the NP460hTert cells, and its levels remained unchanged following EBV infection. Given that EBV infection can reduce MAOA expression even in the pre-malignant NPE cells, it suggests that such regulation is an early and crucial event in the pathogenesis of undifferentiated NPC. Nonetheless, we noted that MAOA was not reduced in all primary NPC examined, suggesting other carcinogenic mechanisms override the necessity for the loss of MAOA expression.

It is well-recognised that EBV infection alters multiple cellular signalling cascades that drive the progression and development of NPC. Constitutive activation of STAT3 is commonly reported in NPC^33,34^. IL-6 is a potent activator of STAT3 which binds to its cognate receptor on the cells membrane, IL-6R. Upon binding, Janus tyrosine kinases (JAKs) are activated and subsequently induce phosphorylation and dimerization of STAT3^35^. Elevated levels of IL-6 were reported in sera of NPC patients and overexpression of IL-6R in primary NPC tissues^25,36^. Further, *in vitro* studies have shown that EBV infection of NPE cells led to the activation of the STAT3 pathway through IL-6/IL-6R signalling and this resulted in increased growth and invasive properties of NPE cells^25,37^. Down-regulation of MAOA by IL-6 or IL-6/IL-6R signalling has been inferred from a limited number of studies. The expression of MAOA is inhibited in chlorangiocarcinoma through IL-6 signalling via regulating the balance between SP-1 transcriptional activity (a positive regulator of MAOA) and its inhibitor, R1 repressor^12^. In breast cancer cells, activation of IL-6/IL-6R signalling suppressed MAOA expression in hypoxic environment^14^. We are the first to demonstrate that IL-6/IL-6R signalling down-regulates MAOA via the activation of STAT3, an effect that is likely attributable to EBV infection. Although we found that addition of IL-6 enhanced the migration of NP460hTert cells (Supplementary Fig. S3), we did not observe any increases in IL-6 levels following EBV infection of NP460hTert and HONE1 cells (data not shown). Our data suggest that the down-regulation of MAOA by IL-6/IL-6R signalling in NP460hTert/EBV cells was mediated by the EBV-induced IL-6R overexpression.

Aberrant DNA methylation is one of the major epigenomic alterations that affect cancer development, leading to transcriptional silencing of tumour suppressor genes. The unique methylome of NPC and EBV-associated gastric cancer (EBVaGC) suggests the existence of an EBV-specific “epigenetic signature”^30,38^. Several EBV latent proteins can regulate multiple components of the cellular CpG methylation machinery, including DNA methyltransferases (DNMTs), histone modifiers and chromatin remodelers^26^. LMP1 can upregulate the transcripts of DNMT1, DNMT3a and DNMT3b through the activation of JNK signalling in epithelial cells^39^. In EBVaGC, LMP2A is thought to induce the wide-spread hypermethylation by up-regulating cellular DMNT1 expression through the activation of STAT3 signalling^40^. The ability of EBNA1 to interfere with HDAC3 was also evident in Burkitt lymphoma cells^41^. In the present study, we show that MAOA is down-regulated by EBNA1 and LMP2A in HONE1 cells and it is possible that these EBV genes modulate the cellular methylation machinery that results in MAOA reduction.

In conclusion, we report for the first time that MAOA is a putative tumour suppressor gene in NPC and its expression is regulated by EBV infection. Our data highlight the central role of EBV in the pathogenesis of NPC.

## Materials and Methods

### Cell lines and tissue samples

A series of cell lines that included three immortalized NPE cell lines (NP69, NP361hTert, NP460hTert) and eight NPC-derived cell lines, seven of which are EBV-negative (CNE1, CNE2, HK1, HONE1, SUNE1, TW01 and TW04) and one is EBV-positive (C666-1), were used in this study. NPC cells stably infected with a recombinant EBV (Akata strain) or expressing individual EBV-encoded latent genes were generated as previously described^42^. Archival FFPE NPC and non-malignant nasopharyngeal tissues were obtained from the Sime Darby Medical Centre Subang Jaya, Malaysia. All samples were non-keratinising EBER-positive NPC. Ethical approval for this study was obtained from the Independent Ethics Committee, Sime Darby Healthcare, Malaysia (Ref # 201206.2).

### Quantitative reverse transcription PCR (RT-qPCR)

Expression of MAOA and IL-6R was examined by RT-qPCR as previously described^43^. Total RNA was extracted using a RNeasy Mini Kit (Qiagen, UK) and subjected to reverse transcription using High-Capacity cDNA Reverse Transcription kit (Applied Biosystems, USA). Quantitative PCR was performed in triplicate using a QuantiNova SYBR Green PCR Kit (Qiagen) and an ABI Prism 7000 Sequence Detection System (Applied Biosystems). The primers used are listed in Table S1. GAPDH was amplified in the same reaction to serve as an internal control for normalization. Fold changes in gene expression were measured using the comparative threshold cycle method (ΔΔCt).

### Immunohistochemistry

Expression of MAOA proteins in primary NPC tissue samples was determined by immunohistochemistry using the DAKO REAL EnVision Detection System (DakoCytomation, Denmark) as described previously^42^. Anti-MAOA rabbit monoclonal antibody (Abcam, UK) was used at 1:150. Non-neoplastic tonsils demonstrating reactive lymphoid hyperplasia were used as positive controls. Immunohistochemical staining was evaluated semi-quantitatively using the H-score method. The percentage of tumour corresponding to an ordinal intensity value (0 = none, 1 = weak, 2 = moderate, 3 = strong) was assigned using whole sections. The H-score was defined as the sum of the percent of tumour cells staining multiplied by the intensity level, resulting in a score ranging from 0 (no staining in any of the tumour cells) to 300 (strong staining in all tumour cells). Where available, H-scoring was also undertaken for intra-sectional normal surface respiratory epithelium.

### Western blotting

Protein expression of MAOA and STAT3 was examined by Western blotting as previously described^43^. Cells were lysed in ice-cold NP40 lysis buffer [150 mM NaCl, 1% IGEPAL CA-630, 50 mM Tris-HCl (pH 8.0)] containing protease inhibitors (cocktail set III; Calbiochem, Merck Millipore) and phosphatase inhibitors (Halt phosphatase inhibitor cocktail; Thermo Scientific, USA). Samples containing equal amounts of protein were separated under reducing conditions using SDS-PAGE and the proteins transferred to polyvinylidene difluoride membranes (Millipore, UK). The primary antibodies used in this study were anti-MAOA (1:1000; Abcam, UK), anti-phospho-STAT3 (Tyr705; 1:1000; Cell Signaling), anti-total STAT3 (1:1000; Cell Signaling) and anti-β-actin (1:5000; Sigma-Aldrich, USA). Bound antibodies were detected with peroxidase conjugated secondary antibodies and Enhanced Chemiluminescence reagents (Advansta, USA).

### MAOA overexpression and knockdown

The expression plasmid carrying the coding region of MAOA, pEZ-lv105/MAOA, was kindly provided by Prof Zhi-Gang Zhang (Shanghai Cancer Institute Shanghai Jiao Tong University, Shanghai, China). Cells at 50% confluency were transfected with the pEZ-lv105/MAOA or the empty vector at a 3:1 FuGENE HD transfection reagent (Promega, USA):DNA ratio. Cells were used in Transwell migration assays 24 hours post-transfection. To knockdown MAOA, pre-designed RNAi SMART pool reagents for MAOA (L-009369–00) and non-targeting random siRNAs were obtained from Dharmacon, USA and used at 25 nM. 5 × 10^5^ cells were seeded into 100mm^2^ dishes, cultured for 16 hours and then transfected with the relevant siRNA using DharmaFECT 1 transfection reagent (Dharmacon, USA). Cells were used in Transwell migration assays 48 hours post-transfection.

### Transwell migration assays

Migration assays were carried out using fibronectin-coated (10 µg/ml) polycarbonate filters (8 µm pore size, Transwell, Corning). Cells were treated with mitomycin C for two hours prior to the experiments. 5 × 10^4^ cells were seeded into the upper chamber and allowed to migrate for 19 hours in the presence of FBS (Gibco, USA) or IL-6 (Promokine, Germany) in the lower chamber. Migrated cells were stained with 0.1% crystal violet and counted in five random fields.

### Methylation-specific PCR (MSP) and bisulphite sequencing (BSP)

The protocols and DNA samples used for the MSP analysis have been previously described^27–30^. The modified DNA was amplified by PCR with methylated primers (Table S1). Universal methylated DNA was used as positive controls. To re-activate MAOA expression, SUNE-1 cells at 30% confluence were treated with the demethylation agent 5-aza-2′deoxycytidine (5-aza-dC; Sigma-Aldrich, USA) for 4 days and/or a histone deacetylation inhibitor, Trichostatin A (TSA; Sigma-Aldrich) for the last 24 hours. For bisulphite sequencing, The DNA samples were subjected to bisulphite modification using the EZ DNA Methylation-Lightning Kit (Zymo Research, USA). A total of 12 CpG sites spanning the start codon of the *MAOA* promoter were analysed by bisulphite sequencing with primers designed using the MethPrimer software (Table S1).

### Statistical analyses

All statistical analyses were performed using GraphPad Prism Version 5 (GraphPad Software Inc., USA). Statistical differences between experimental groups were evaluated by Student’s t-test or one-way analysis of variance (ANOVA)/Dunnett’s test. *p*-values <0.05 were regarded as significant.

## Supplementary information

Supplementary information.
