# Supplementary material for: Monoamine oxidase A is down-regulated in EBV-associated nasopharyngeal carcinoma
Source: Sci Rep. 2020 Apr 9;10:6115. doi: 10.1038/s41598-020-63150-0 (PMC7145851; doi:10.1038/s41598-020-63150-0)
Supplement: Supplementary file 1 — Supplementary information. [file 41598_2020_63150_MOESM1_ESM.pdf]

Monoamine oxidase A is down-regulated in EBV-associated nasopharyngeal carcinoma

Hui Min Lee[1#], Alice Pei Eal Sia[1#], Lili Li[2], Hans Prakash Sathasivam[3], Melissa Sue Ann Chan[1], Pathmanathan Rajadurai[4], Chi Man Tsang[5,6], Sai Wah Tsao[5], Paul G Murray[7,8], Qian Tao[2], Ian C Paterson[1,9], Lee Fah Yap[1*]

[1]Department of Oral and Craniofacial Sciences, Faculty of Dentistry, University of Malaya, Kuala Lumpur, Malaysia.
[2]Cancer Epigenetics Laboratory, Department of Clinical Oncology, State Key Laboratory of Translational Oncology, Sir YK Pao Center for Cancer and Li Ka Shing Institute of Health Sciences, The Chinese University of Hong Kong, Hong Kong.
[3]Cancer Research Centre, Institute for Medical Research, Shah Alam, Malaysia.
[4]Sime Darby Medical Centre Subang Jaya, Subang Jaya, Malaysia.
[5]School of Biomedical Sciences and Center for Cancer Research, Li Ka Shing Faculty of Medicine, The University of Hong Kong, Hong Kong.
[6]Department of Anatomical and cellular Pathology and State Key Laboratory of Translational Oncology, The Chinese University of Hong Kong, Hong Kong.
[7]Health Research Institute, University of Limerick, Limerick, Ireland.
[8]Institute of Immunology and Immunotherapy, University of Birmingham, Birmingham, United Kingdom.
[9]Oral Cancer Research and Coordinating Centre, Faculty of Dentistry, University of Malaya, Kuala Lumpur, Malaysia.

Supplementary Figure S1

Figure 1B

Images with protein marker

Images without protein marker

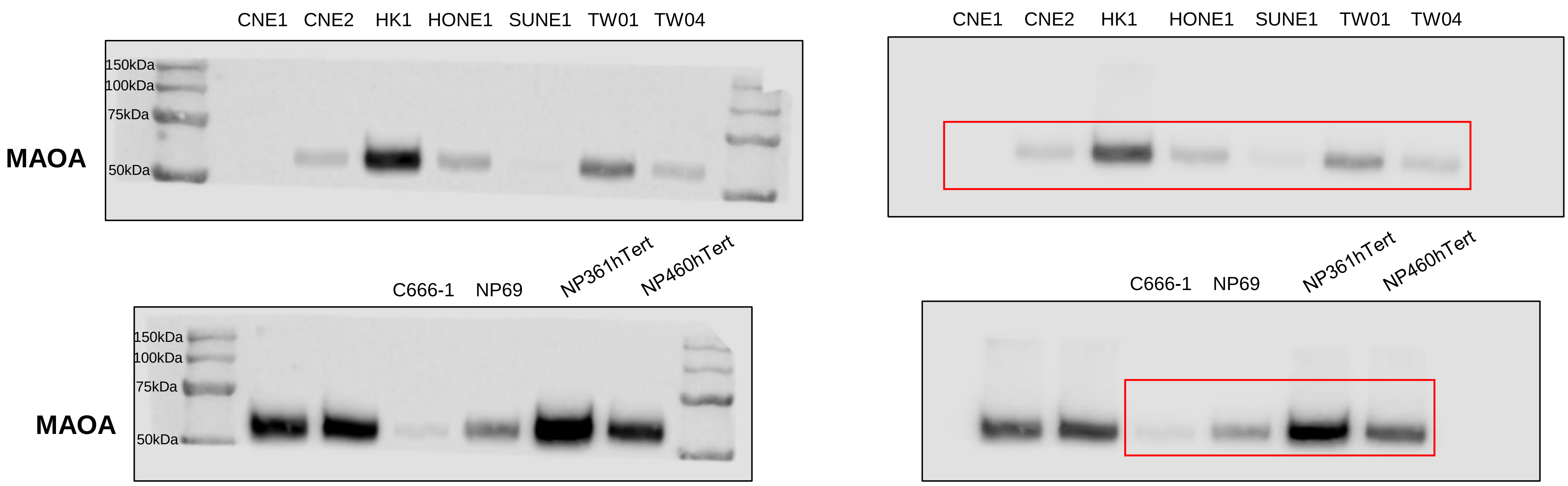

Figure 1B

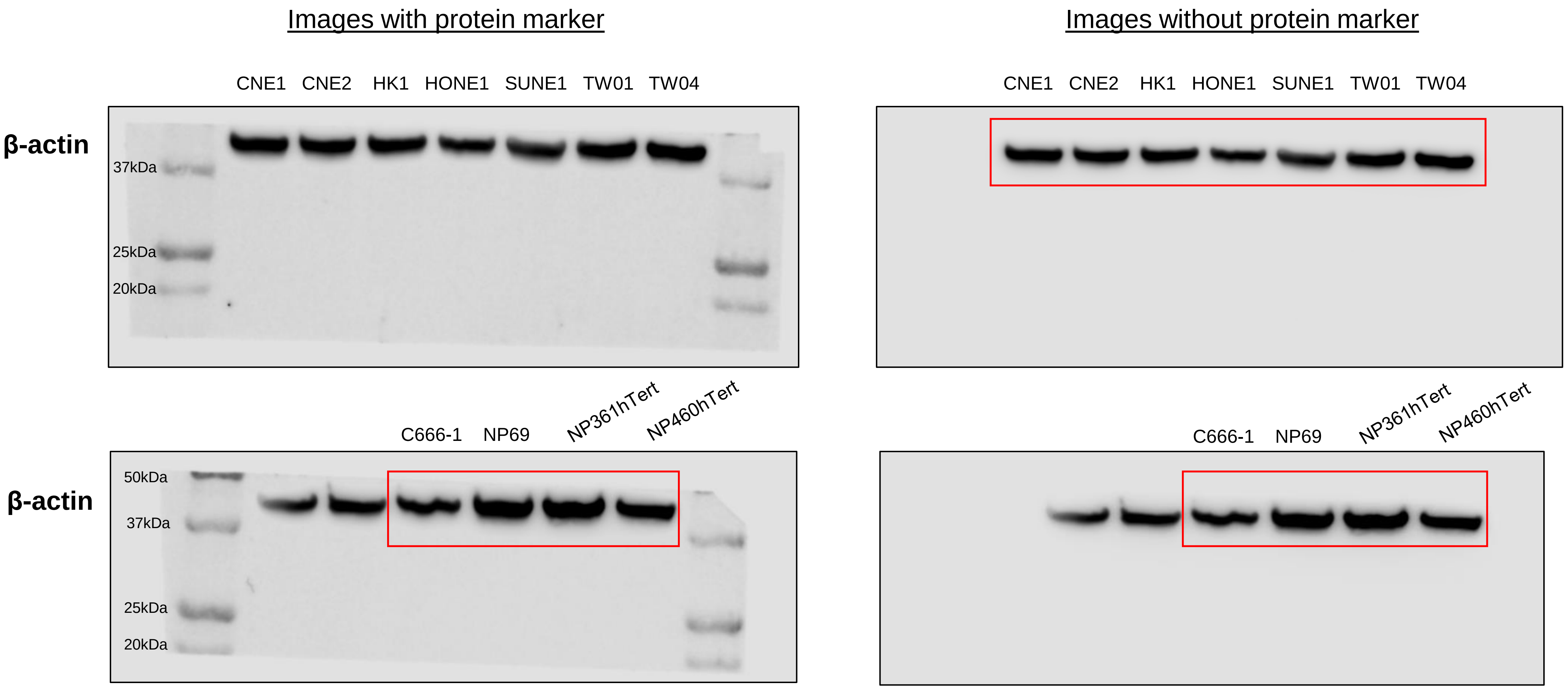

Images with protein marker
Images without protein marker
CNE1 CNE2 HK1 HONE1 SUNE1 TW01 TW04
β-actin
37kDa
25kDa
20kDa
CNE1 CNE2 HK1 HONE1 SUNE1 TW01 TW04
C666-1 NP69 NP361hTert NP460hTert
β-actin
50kDa
37kDa
25kDa
20kDa
C666-1 NP69 NP361hTert NP460hTert

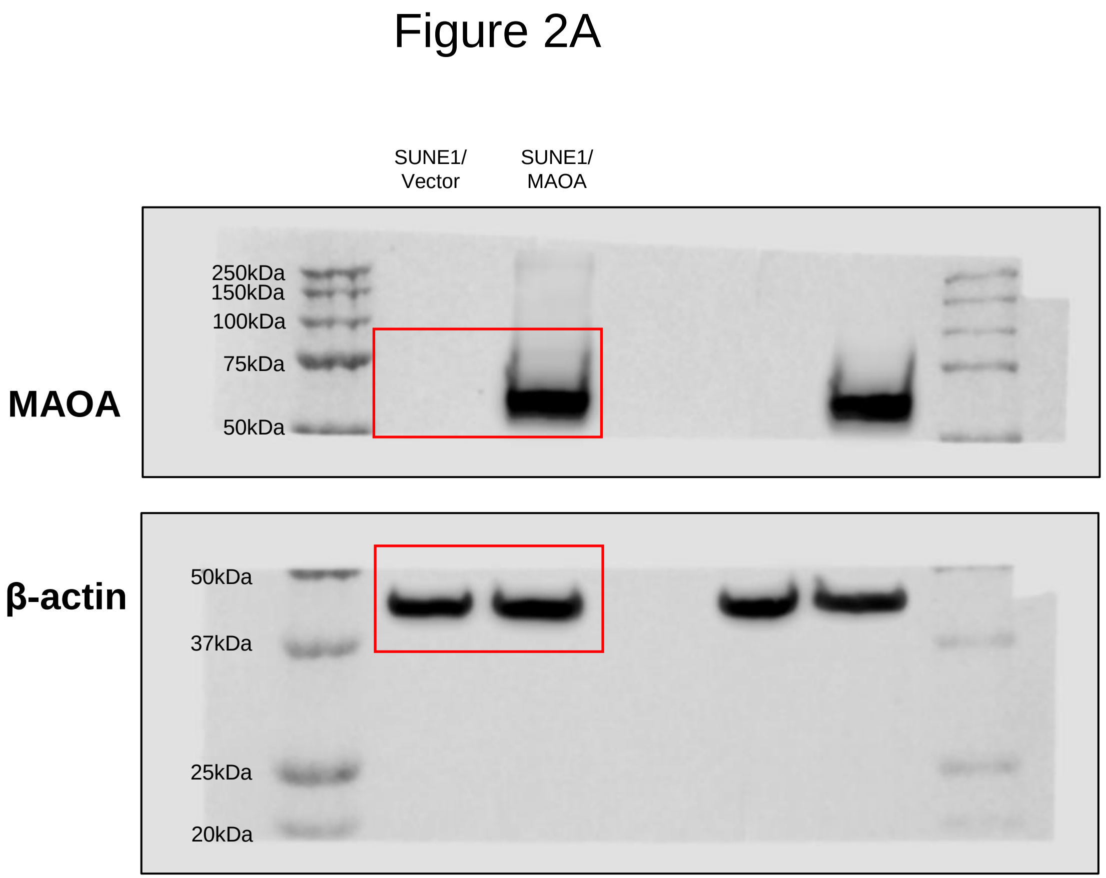
Figure 2A
SUNE1/
Vector
SUNE1/
MAOA
MAOA
250kDa
150kDa
100kDa
75kDa
50kDa
β-actin
50kDa
37kDa
25kDa
20kDa

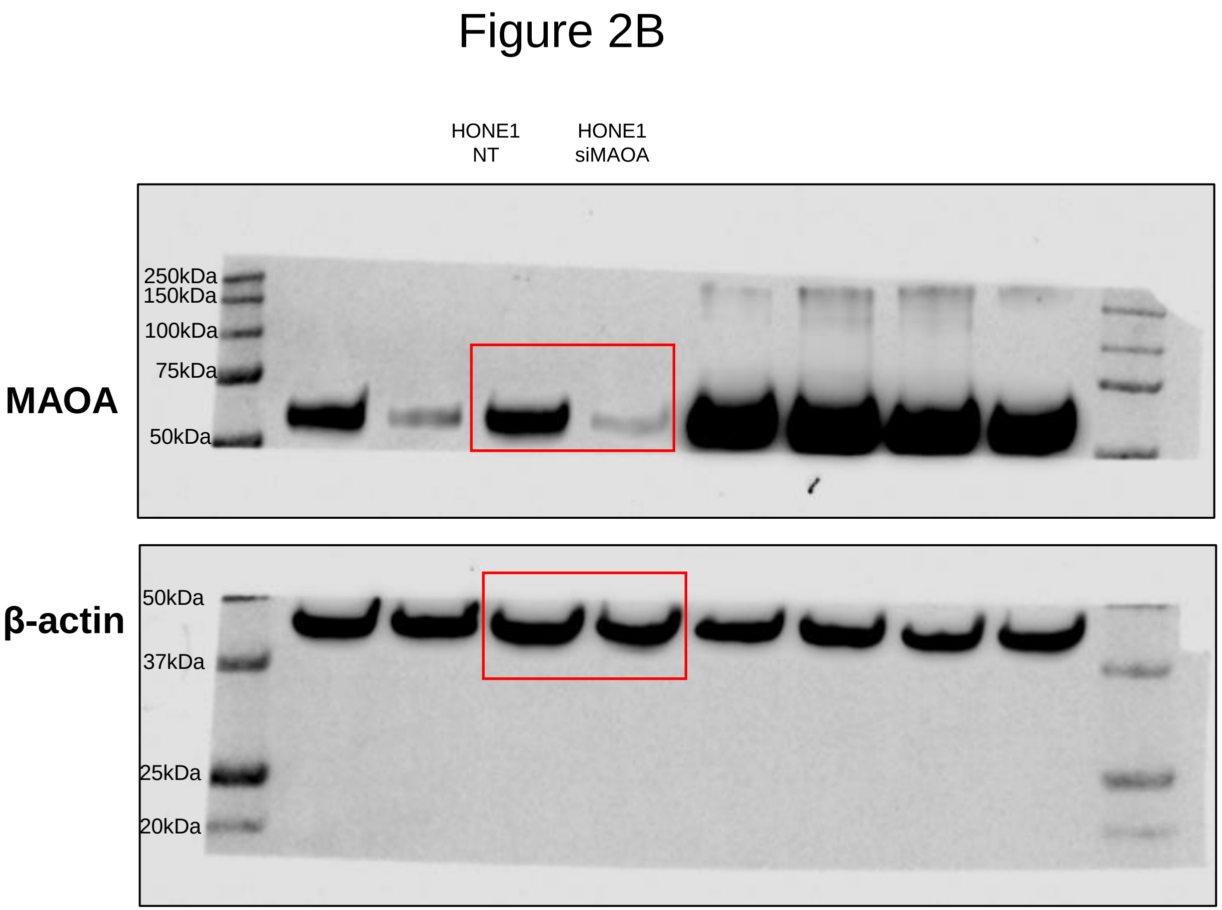
Figure 2B
HONE1
NT
HONE1
siMAOA
MAOA
250kDa
150kDa
100kDa
75kDa
50kDa
β-actin
50kDa
37kDa
25kDa
20kDa

Figure 3B

Images with protein marker

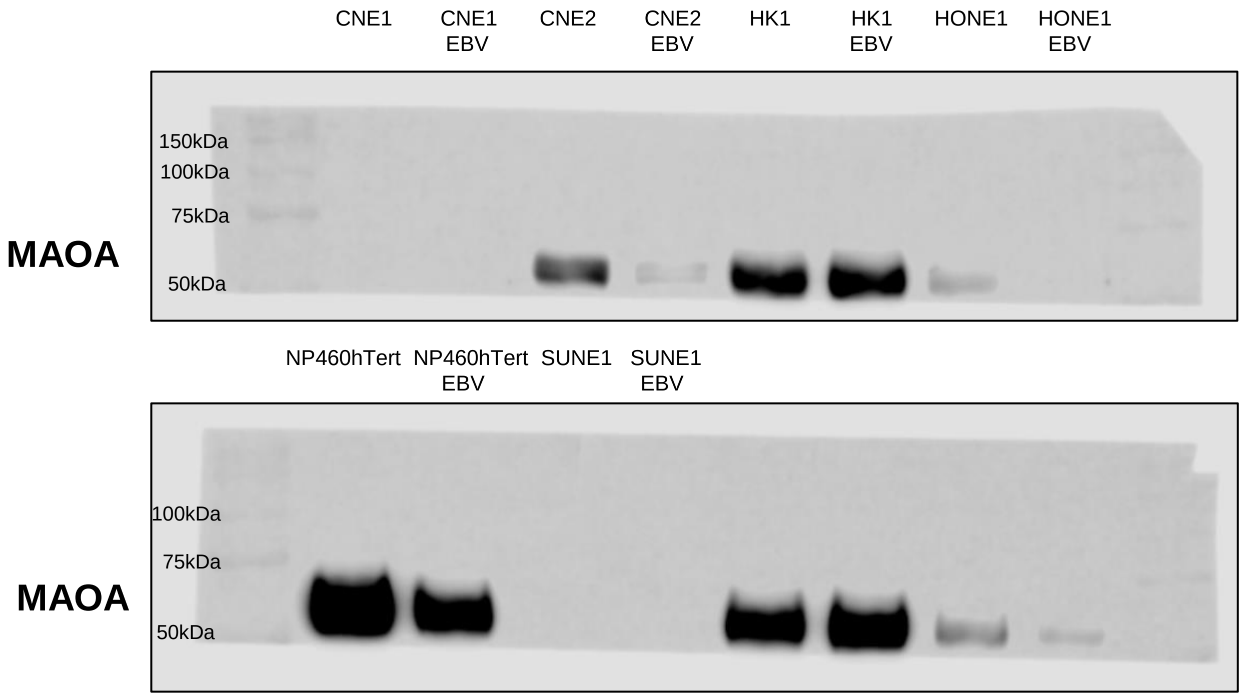

Images without protein marker

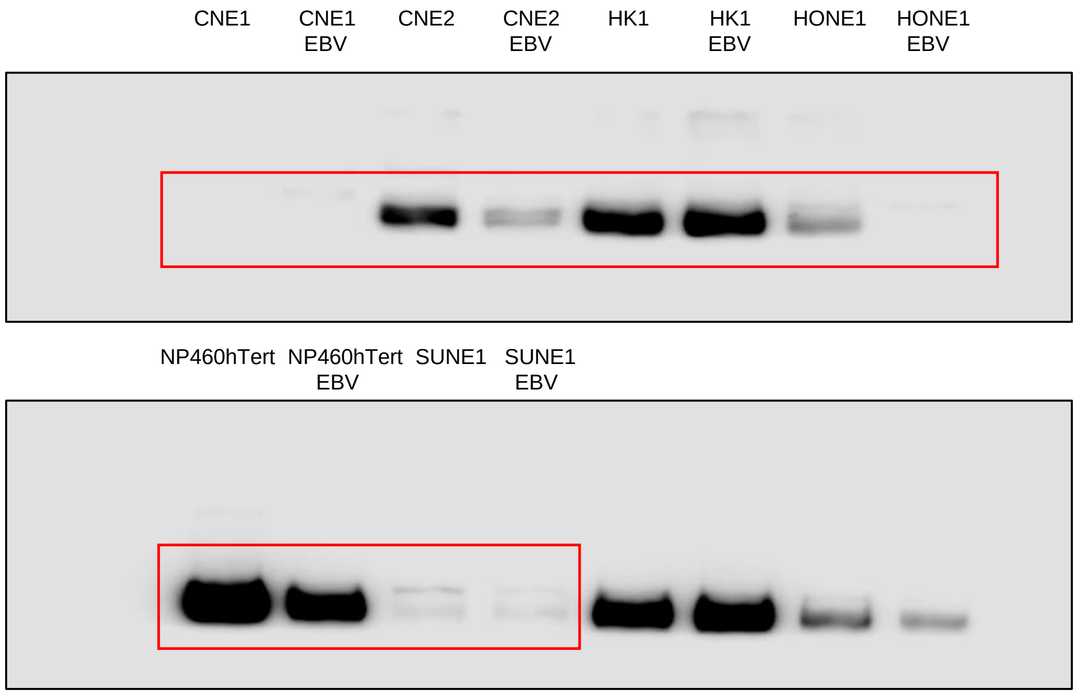

Figure 3B

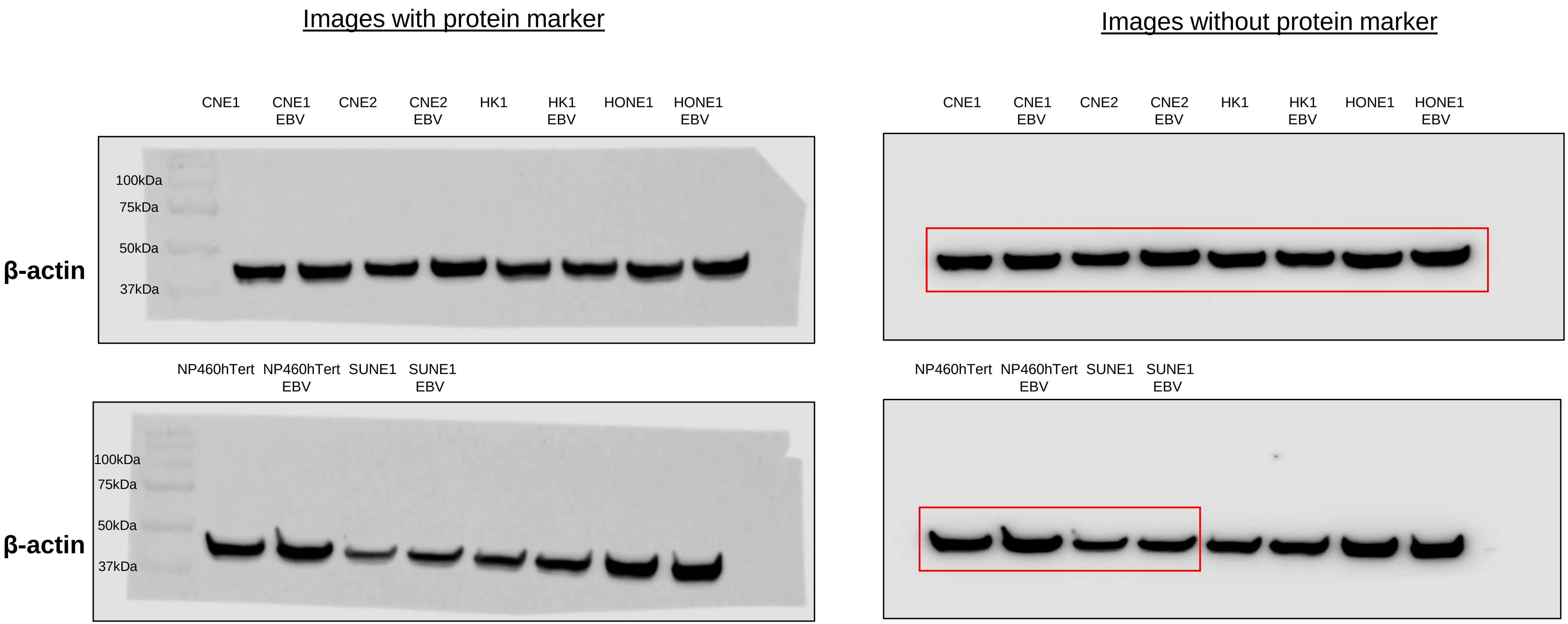
Images with protein marker
Images without protein marker
CNE1
CNE1 EBV
CNE2
CNE2 EBV
HK1
HK1 EBV
HONE1
HONE1 EBV
100kDa
75kDa
50kDa
37kDa
β-actin
NP460hTert
NP460hTert EBV
SUNE1
SUNE1 EBV

Figure 3C

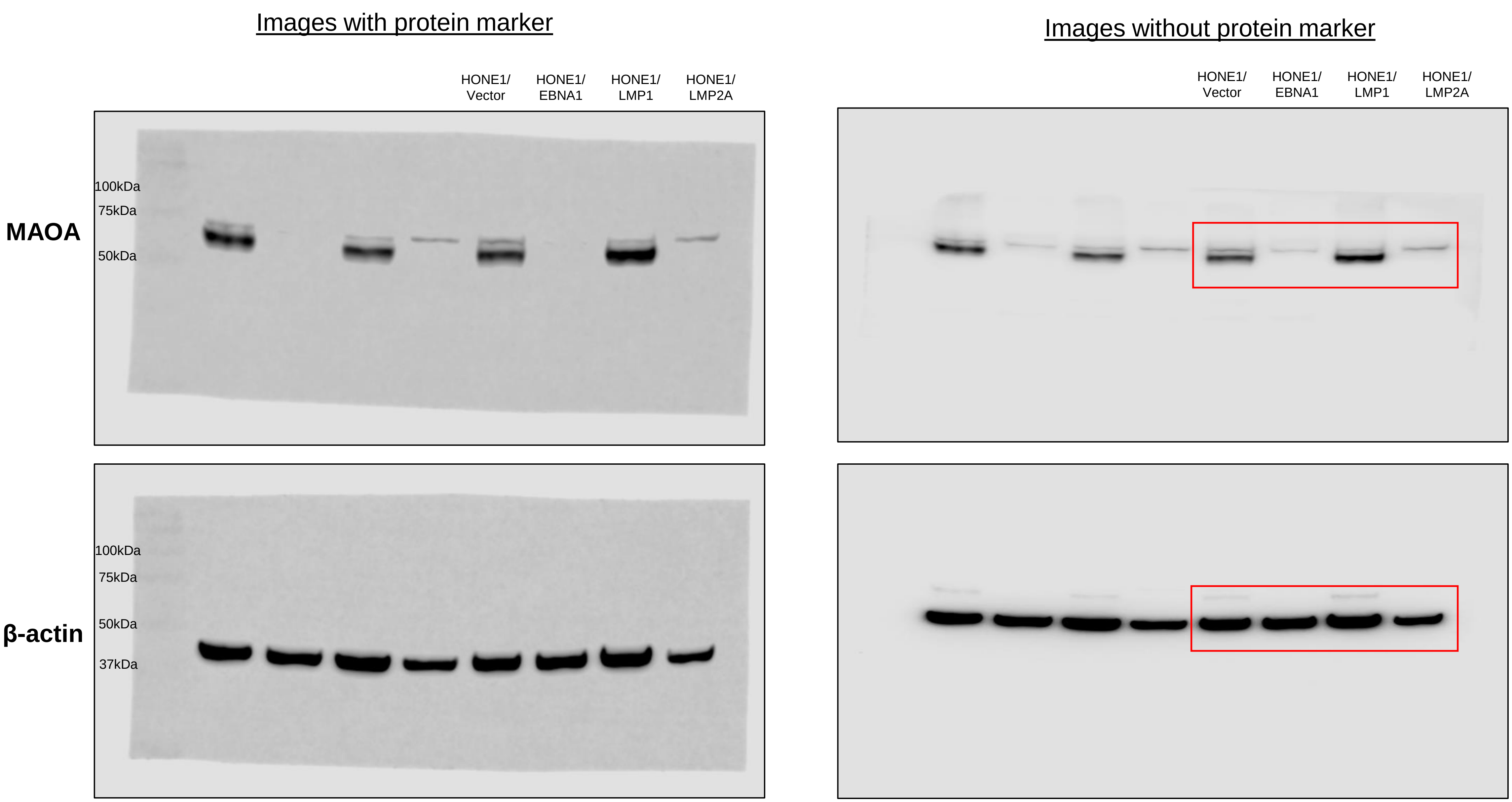
Images with protein marker
Images without protein marker
HONE1/ Vector
HONE1/ EBNA1
HONE1/ LMP1
HONE1/ LMP2A
MAOA
100kDa
75kDa
50kDa
β-actin
100kDa
75kDa
50kDa
37kDa
HONE1/ Vector
HONE1/ EBNA1
HONE1/ LMP1
HONE1/ LMP2A

Figure 4B

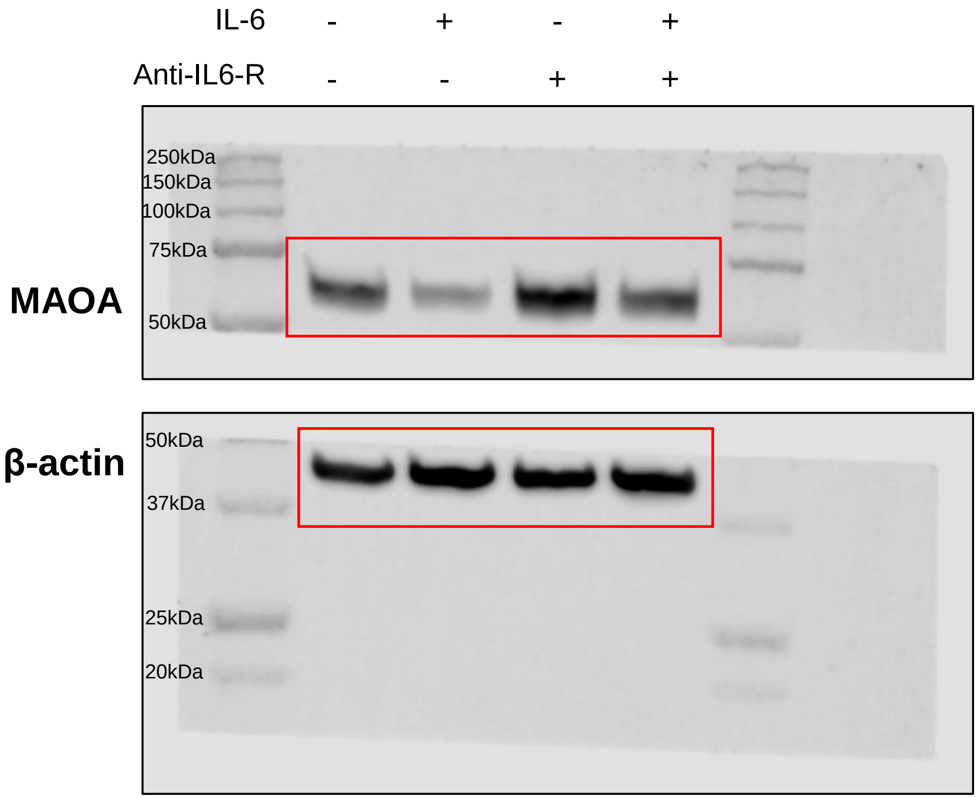
IL-6 - + - +
Anti-IL6-R - - + +
MAOA
250kDa
150kDa
100kDa
75kDa
50kDa
β-actin
50kDa
37kDa
25kDa
20kDa

Figure 4C

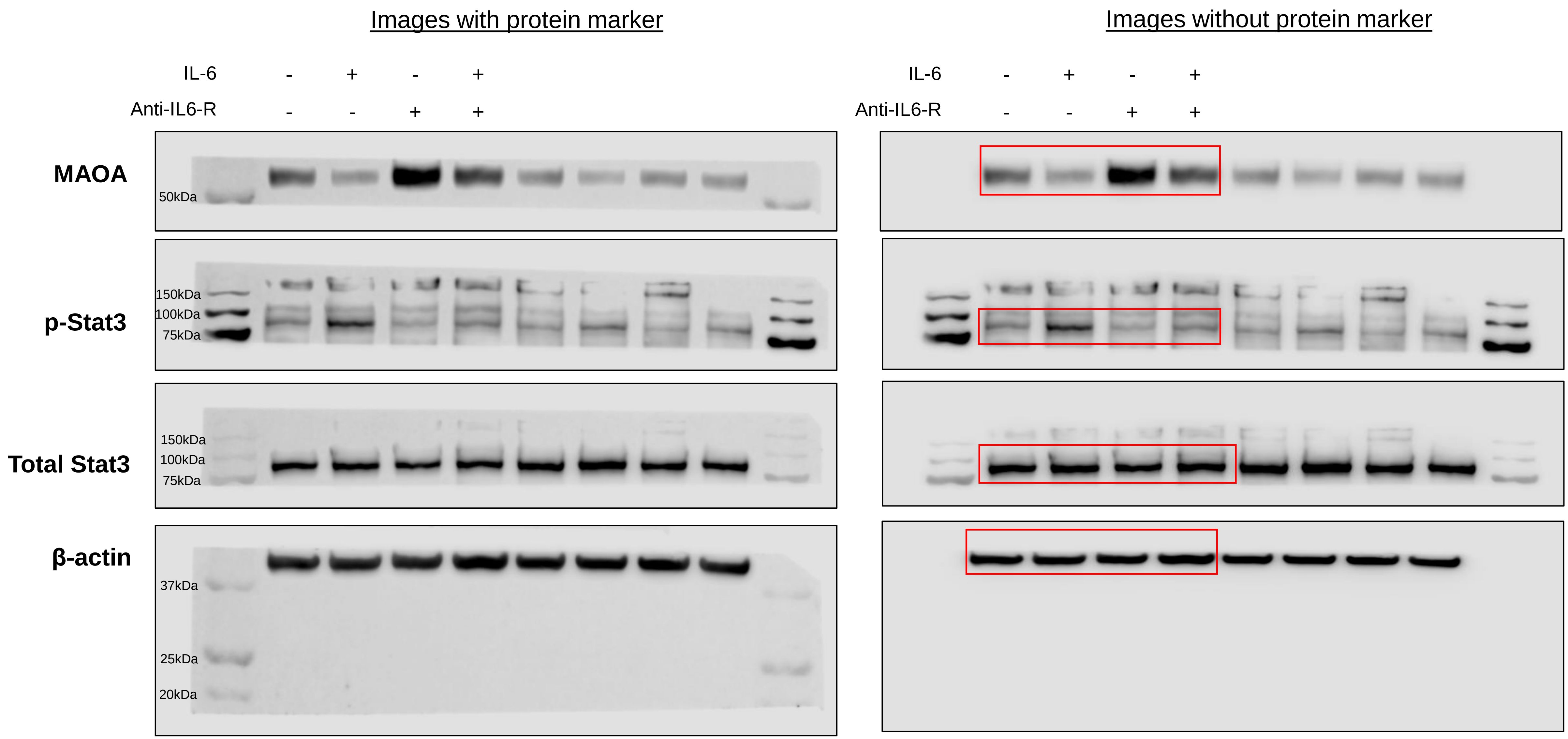
Images with protein marker
Images without protein marker
IL-6
- + - +
Anti-IL6-R
- - + +
MAOA
50kDa
p-Stat3
150kDa
100kDa
75kDa
Total Stat3
150kDa
100kDa
75kDa
β-actin
37kDa
25kDa
20kDa

Figure 4D

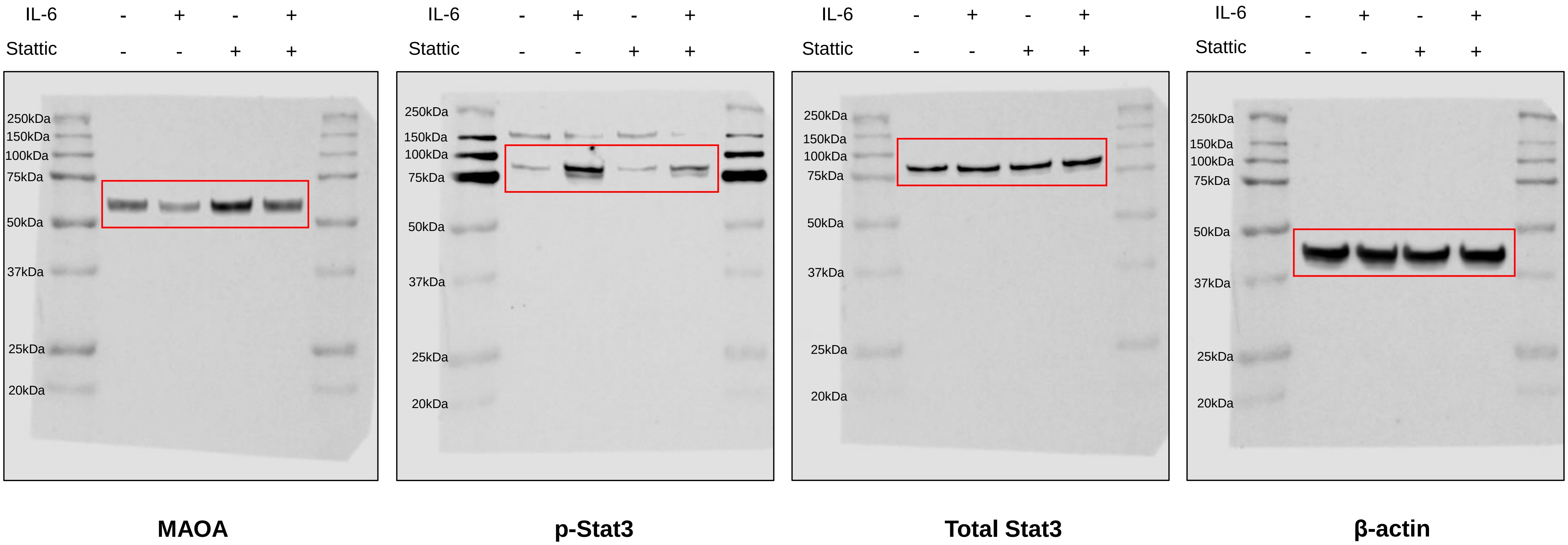

IL-6 - + - +
Stattic - - + +
250kDa
150kDa
100kDa
75kDa
50kDa
37kDa
25kDa
20kDa
MAOA
p-Stat3
Total Stat3
β-actin

Figure 5B

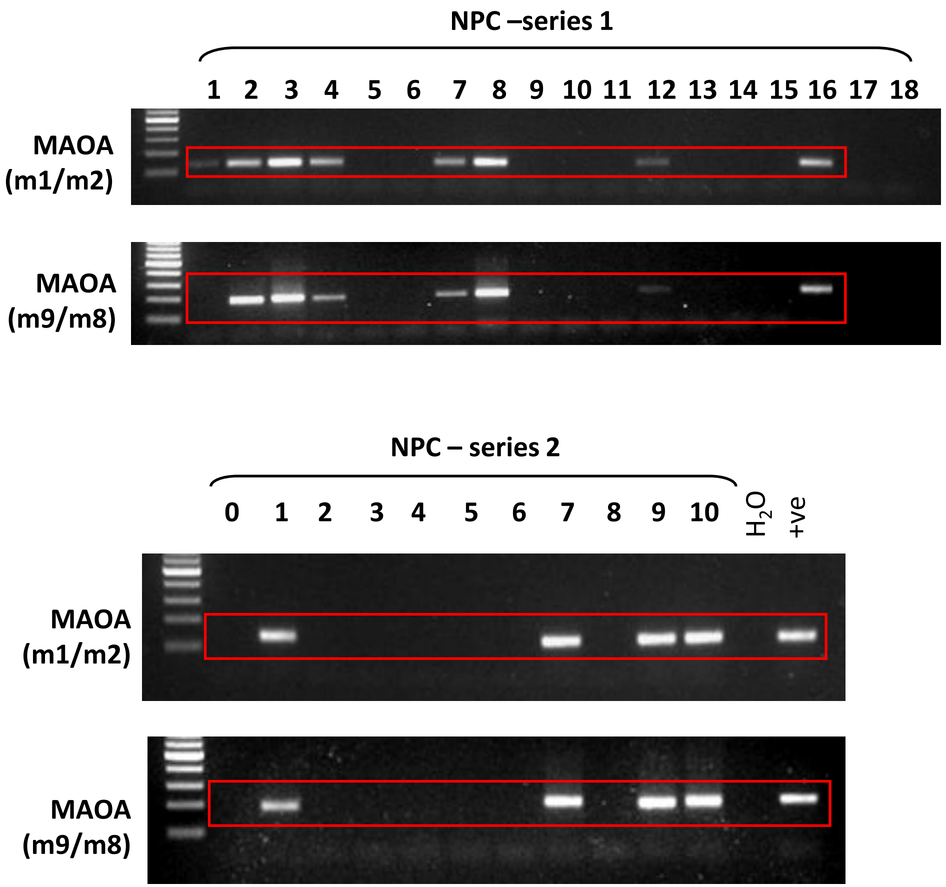

Figure 5C

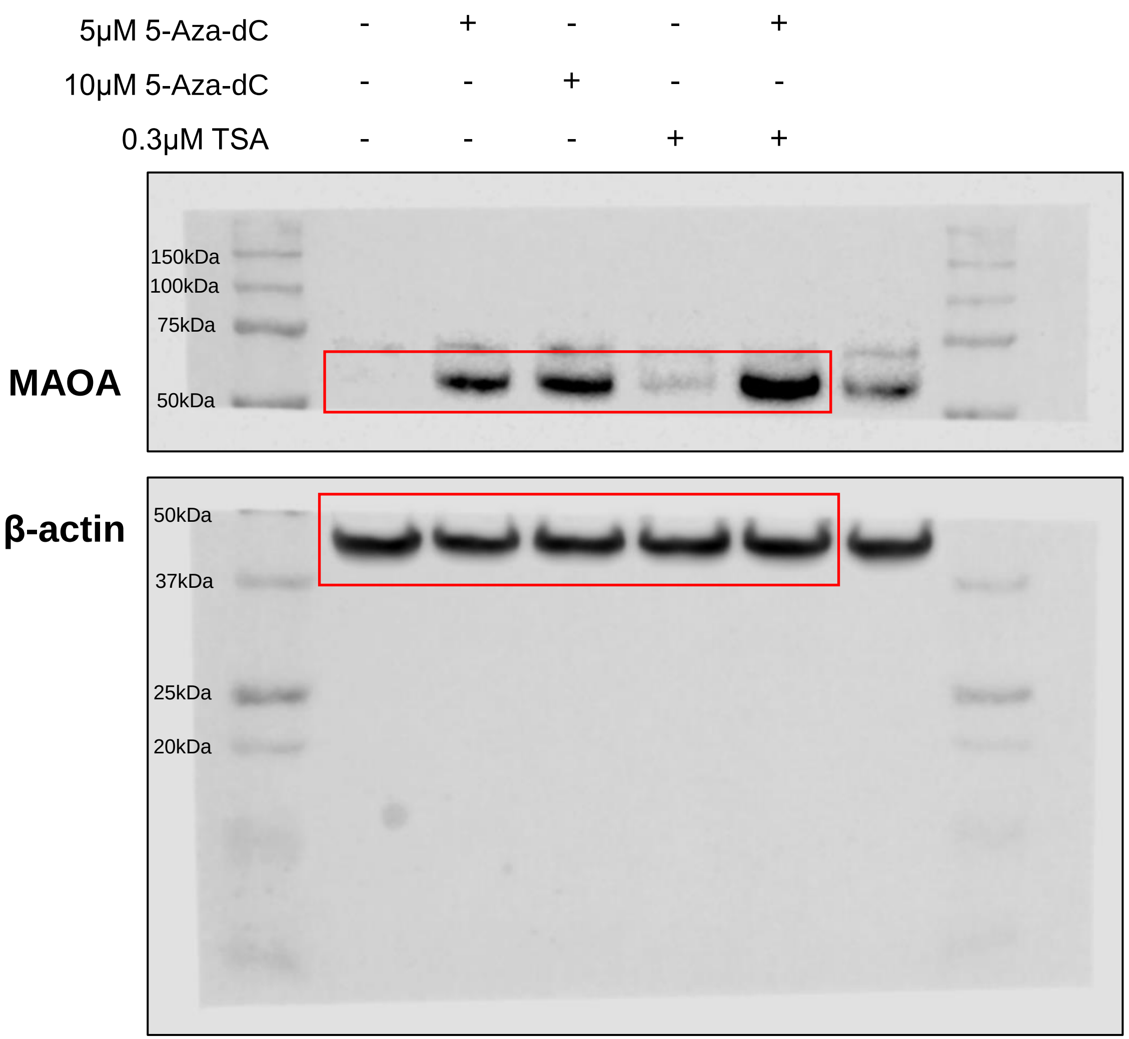

Supplementary Figure S1 Uncropped scans of western blots and electrophoresis gels

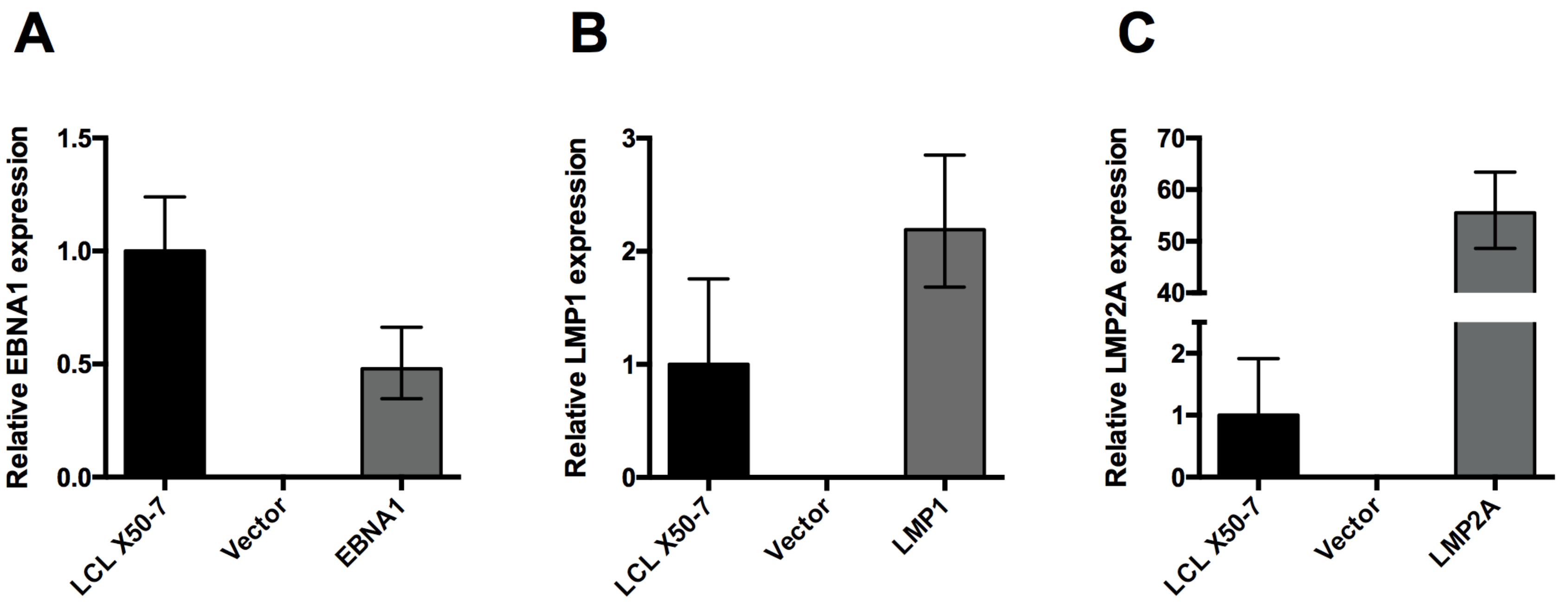

Supplementary Figure S2 RT-qPCR confirmed the mRNA expression of EBNA1, LMP1 and LMP2A in HONE1 cells transfected with specific EBV latent genes. LCL X50-7 was used as a positive control (normalised to 1).

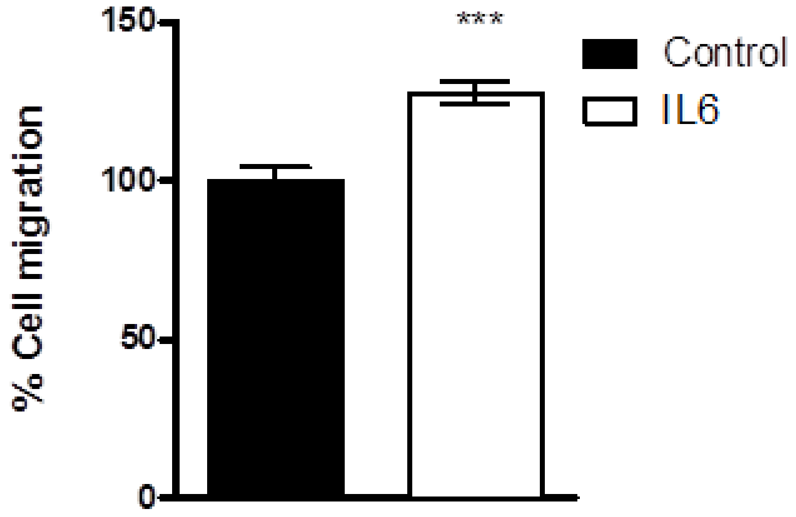

Supplementary Figure S3 Exogenous addition of IL-6 (50ng/ml) increased the migration of NP460hTert cells.

Table S1. Sequences of primers for RT-qPCR, methylation-specific PCR (MSP) and bisulphite sequencing (BSP).

**RT-qPCR**	
MAOA	(F) 5’CTGATCGACTTGCTAAGCTAC<br>(R) 5’ATGCACTGGATGTAAAGCTTC
IL-6R	(F) 5’CCCCATCCCTGACGACAAA<br>(R) 5’TGGGACTCCTGGGAATACTG
**MSP**	
MAOA-m1/m2	(F) 5’ TAGTGGTATCGTTCGGTTAC<br>(R) 5’ ACCAACCCCGAACTCCCG
MAOA-m9/m8	(F) 5’ TAAAACCCGTCGAAATCTACG<br>(R) 5’ TGACGGTTTTCGTTTCGTTTC
**BSP**	
MAOA	(F) 5’TAGAAGGGTTTTTTTATTTTTTGT<br>(R) 5’TATAAATTCCCCTACCCCTCACTA
